# Single-Port Surgery in Inflammatory Bowel Disease: A Review of Current Evidence

**DOI:** 10.1007/s00268-016-3509-y

**Published:** 2016-04-19

**Authors:** E. Joline de Groof, Christianne J. Buskens, Willem A. Bemelman

**Affiliations:** Department of Surgery, Academic Medical Center, PO Box 22660, 1100 DD Amsterdam, The Netherlands

## Abstract

The majority of patients with Crohn’s disease and up to 35 % of patients with ulcerative colitis will ultimately require surgery during the course of their disease. Over the past few years, surgical techniques and experience in minimal invasive surgery have evolved resulting in single-incision laparoscopic surgery. The aim of this approach is to diminish the surgical trauma by reducing the number of incision sites. This review discusses the benefits and disadvantages of single-port surgery in various procedures in patients with inflammatory bowel disease (IBD). Short-term postoperative results, functional outcome, and costs available in the literature will be discussed. Single-port surgery in IBD has several benefits when compared to multi-port laparoscopic surgery. By using fewer incisions, a potential reduction of postoperative pain with less morphine use can be accomplished. In addition, accelerated postoperative recovery can result in a shorter hospital stay. Furthermore, a superior cosmesis can be reached with placement of the port at the future ostomy site or at the umbilicus. Literature on single-port surgery in IBD consists mainly of case series and a few matched case series. These studies demonstrated that single-port surgery seems to be a safe and feasible approach for the surgical treatment of IBD patients.

## Introduction

Most inflammatory bowel disease (IBD) patients are initially treated with medical therapy [1]. Although nowadays there seem to be a trend of reduced surgical interventions due to the optimization of medical therapy in the anti-TNF era, the majority of patients with Crohn’s disease (CD) and up to 35 % of patients with ulcerative colitis (UC) still require intestinal resection during the course of their disease [2]. Therefore, repetitive surgery is more a character of the disease rather than a complication due to recurrence or progression of the disease [3].

Established indications for surgery in IBD include therapy refractory disease, unacceptable medical therapy side-effects and, in patients with CD, a perforation or obstruction. Procedures depend on the localization and extension of the disease and vary from ileocecal resections for localized disease in CD to restorative proctocolectomy with ileal pouch-anal anastomosis (IPAA) for extensive therapy refractory UC.

Multi-port laparoscopic surgery for IBD was first reported in the early 1990s [4]. Since then experience has evolved and more complex laparoscopic procedures were performed for both CD and UC. There is an abundance of literature demonstrating that laparoscopic small and large bowel resections are safe and efficacious, resulting in reduced postoperative complications, earlier recovery, and improved cosmesis [5, 6]. Therefore, laparoscopy is considered to be an important surgical tool in the minimally invasive treatment of IBD patients.

Single-incision laparoscopic resection in colorectal surgery was first described in 2008 by Remzi and Bucher [7, 8]. The aim of this approach is to diminish the surgical trauma by reducing the number of incision sites. There are various ports available with specific benefits and disadvantages. The Triport+ (Olympus) was the first available access system approved by the U.S. Food and Drug Administration (FDA). The introducer tool has a blunt tip allowing safe and easy introduction into the abdominal cavity. The retraction sleeve provides wound protection and streamlines specimen removal. Some other available devices are the QuadPort+ (Olympus), which has five instrument ports, allowing major abdominal surgery. The GelPOINT (Applied Medical), with a fixation ring that acts as a both a wound retractor and protector. The SILS^TM^ Port multiple access port (Covidien) that was designed with adjustable cannulas within a malleable blue port allowing surgeons to use multiple instruments with maximal maneuverability. The Uni-X (Pnavel Systems), a funnel-shaped device with a shorter tunnel for a wide range of motion. For transanal access the GElPOINT Path (Applied Medical) can be used.

Good fixation, the ability to take out the specimen without losing exposure and easier triangulation are all important aspects that contribute to a successful procedure. Furthermore, in a time where cosmesis is considered to be of increasing importance, less invasive surgery with a more favorable postoperative appearance when compared to multi-port or open surgery, is preferred by the patients [9–11].

Recently, a new application of single-port surgery has been adopted in IBD surgery. A hybrid between transanal endoscopic microsurgery (TEM) and single-port laparoscopy, where a transanal multichannel port is used combined with conventional laparoscopic instruments. The improved visualization achieved with this so-called transanal minimally invasive surgery (TAMIS) technique is very promising for minimal invasive completion proctectomy.

So far, the literature on single-incision laparoscopic surgery for IBD is limited. Randomized controlled trials (RCTs) comparing single-port with multi-port laparoscopic surgery are lacking and so far, mainly case series were published.

To obtain an overview of the literature Pubmed and EMBASE were searched using the following search terms: ‘inflammatory bowel disease,’ ‘Crohn’s disease,’ ‘ulcerative colitis,’ ‘single port,’ ‘single incision,’ and ‘TAMIS.’ Review articles, case series and comparative studies were used when available.

This review discusses the benefits and disadvantages of single-port laparoscopic surgery in various procedures in patients with CD and UC. Short-term postoperative results, functional outcome, and costs will be discussed.

## Crohn’s disease

### Ileocecal resection

Ileocecal resection for localized terminal ileitis is a common procedure in CD patients with a stenosis of the ileum or medical therapy refractory disease. Several studies established that single-port ileocecal resection is a safe procedure [12, 13]. Two comparative matched studies were published up to date. Rijcken et al. performed a matched pair controlled study with 20 single-port procedures and 20 traditional laparoscopic ileocecal resections [12]. No differences in complication rates (overall 20 % in both groups) or conversion rates (5 % versus 10%, *p* = 0.55) were reported for single-port laparoscopy when compared to conventional multi-port laparoscopic surgery. A recent study by Gardenbroek et al., where 21 single-port surgery cases were matched to 42 patients who underwent conventional laparoscopic ileocecal resection, also found no significant difference in conversion, complication, and reoperation rates between the groups [13].

Assessing the postoperative course, patients in the single-port group required less morphine the first postoperative day when compared to patients in the conventional multi-port laparoscopic surgery group [12.5 mg, interquartile range (IQR) 5.0–33.3 versus 28 mg, IQR 15.0–50.0, *p* = 0.012, respectively] [13]. Postoperative pain scores (VAS) did not differ indicating an adequate pain management. Hospital stay was significantly shorter in the single-port group with a median postoperative stay of 4 days (IQR 4–5) when compared to 5 days (IQR 4–6) in the multi-port laparoscopic surgery group. This difference was not observed in the study by Rijcken et al.

Surprisingly, mean operative time was significantly shorter in the single-port group compared to the standard laparoscopy group [137.4 min, standard deviation (SD) 28.4 versus 166.4 min, SD 37.5, *p* = 0.009] [12]. This was reported as well in the paper by Gardenbroek et al., (103.0 min, IQR 94.0–121.0 versus 123.5 min, IQR 100.0–157.0 respectively, *p* = 0.036). However, this probably reflects the overall improvement of laparoscopic skills of the surgeons. It has to be emphasized that both studies reported a comparison based on procedures that have been performed in different points in time with the possible introduction of selection bias. In addition, the shift from hand-sewn anastomoses to stapled anastomoses, which is most indicated nowadays, could have influenced the procedure time [14].

Moftah et al. reported a mean operative time of 120 min (range 80–120) for both primary single-port ileocecal resections and redo single-port ileocolonic resections [15]. In 3 out of 25 (12 %) patients in the primary single-port resection group, conversion was required, where this was 2 out of 6 (33 %) in the redo single-port resection group [15]. The overall median postoperative hospital stay was 6 days (range 3–23).

### (Segmental) colectomy

The extensiveness of the colonic resection depends on the degree of large bowel involvement. Segmental colectomy may be adequate for localized colonic involvement only, where a completion proctectomy may be indicated in patients with extensive, diffuse colorectal disease. In emergency settings, a subtotal colectomy is usually performed with the creation of an ileostomy. The literature on single-port laparoscopic surgery for CD in the emergency setting is scarce.

Two systematic reviews on single-port colonic surgery were published to date [16, 17]. However, only a small number of patients in the included studies were diagnosed with CD.

Makino et al. included three studies (*n* = 88 patients) with overall 11 CD patients [17–20]. Conversion rates in these studies varied from 0 to 10 %. Operative times were similar (medians ranged from 110 to 129 min). Overall, they concluded that in early series of highly selected patients, single-incision laparoscopic colectomy appears to be feasible [17]. Hospital stay varied between the studies.

Fung et al. compared colonic single-port laparoscopy data with a Cochrane review focusing on the short-term outcomes of conventional laparoscopic colonic surgery and four randomized controlled trials addressing laparoscopic colectomy [16]. Indications for surgery included benign as well as malignant disease. Similar median operative times, time to first bowel motion, and hospital stay were observed between the groups. With respect to postoperative pain, conflicting results were reported. Visceral obesity was the main cause of conversion in the studies.

### Transperineal completion proctectomy

Fistula development in CD ranges from 14 to 38 % in population-based estimates and is associated with considerable morbidity rates resulting in a negative impact on quality of life [21]. In specific patients with severe perianal involvement, a transperineal completion proctectomy may be indicated as a last resort option. Recently, a paper by de Nes et al. was published, where a double single-port procedure for transanal intersphincteric proctectomy and abdominal ileorectal anastomosis was described [22]. The perineal phase consisted of dissection in the intersphincteric plane to the pelvic floor and induction of a pneumorectum with close rectal dissection carried distally to the peritoneal reflection. Simultaneously, the loop ileostomy was closed and the ileorectal anastomosis was resected with creation of an endileostomy. There were no postoperative complications.

Single-port laparoscopic completion proctectomy, with a single port at the ostomy site has also been described in the literature [23–25]. However, this regarded a patient with UC, two patients with rectal carcinoma, and a patient with familial adenomatous polyposis. Gaujoux et al. showed that complex procedures such as proctectomy with TME and intersphincteric resection can be performed safely using only two ports in carefully selected patients [23, 24]. Common complications after completion proctectomy are poor perineal wound healing and a persistent presacral sinus (approximately 40 %) [26, 27].

## Ulcerative colitis

### Colectomy

In patients with severe (acute) colitis, a subtotal colectomy with end ileostomy may be indicated, with restorative proctectomy at a later stage when patients are in better condition. So far, literature on single-port laparoscopic colectomy for UC consists only of several case series assessing its feasibility. However, most series consist of a mixed patient population with UC, CD, and FAP patients or patients with colorectal cancer.

Fichera et al. published a case series consisting of nine consecutive patients with medically refractory UC that underwent a single-port laparoscopic total colectomy [28]. The mean operating time was 142 min (SD 23 min) and the mean postoperative length of stay was 5.2 days (SD 1.3 days) [28]. There were no postoperative complications. A study by Vestweber et al. reported on six patients (of which five diagnosed with UC) undergoing a subtotal colectomy [29]. The mean operative time was 223.2 min (range 106–359) and the mean hospital stay was 15.3 days (range 9–31) [29].

For severe acute colitis with patients operated upon in semi-emergency setting, the laparoscopic procedure has also been demonstrated to have several advantages with reduced incidence of postoperative intra-abdominal abscesses [30].

### Proctocolectomy or completion proctectomy with IPAA

Single-port laparoscopic IPAA (for familial adenomatous polyposis) has first been reported in 2010 [31]. Later on, single-port laparoscopic IPAA was used for UC patients as well. So far, the literature is limited to a few case series demonstrating the feasibility and safety of both two- and three-stage procedures [24].

In a study by Geisler et al., 20 patients underwent a proctolectomy or completion proctectomy with an IPAA. Eventually, 11 patients required placement of an additional port for retraction during deep pelvic dissection and placement of a pelvic drain [32]. In a few other (smaller) case series, placement of an additional port was not necessary [24, 33]. Gash et al. published a report on ten UC patients undergoing restorative proctocolectomy and IPAA [33]. The median operative time was 185 min (range 100–381) and the median hospital stay was 3 days (range 2–8) [33]. The median bowel movement frequency at 6 months was four per 24 h. In another study by Geisler et al., a median operative time of 153 min (range 132–278) and a median length of hospital stay of 4 days (range 3–6 days) was reported in five patients undergoing proctocolectomy wih IPAA (of which four with UC) [24]. Vestweber et al. reported a mean operative time of 324 min (range 110–441) and a mean hospital stay of 14.8 days (range 7–21) in nine patients undergoing proctocolectomy with IPAA (of which seven were UC patients) [29].

Despite the limited number of patients in these studies, operative time and length of hospital stay were comparable with conventional laparoscopic surgery.

## (Loop) ileostomy

Creation of a (defunctioning) ileostomy may be required in specific IBD cases to protect the anastomosis or to protect the anorectal region in case of severe perianal disease involvement in CD.

Zhagiyan et al. described single-incision laparoscopic loop ileostomy creation in eight patients of which seven were diagnosed with a Crohn’s proctocolitis [34]. The median duration of surgery was 76 min (range 30–119) and the median length of postoperative hospital stay was 7 days (range 3–15). Postoperative complications consisted of non-operative readmission for ileus and partial small-bowel obstruction, anal dilation, and peristomal cellulitis. Furthermore, two patients developed ischemia of the ostomy, due to bowel edema after laparoscopic manipulation, vascular congestion and a relatively small fascial opening, and required reoperation. These complications rather appeared to be related to the relatively ill group of patients than to the single-incision technique. Although this study consisted of a small sample size, the single-port approach was considered technically feasible and an alternative for traditional laparoscopic ileostomy creation.

## Novel technique: transanal minimally invasive surgery (TAMIS)

The clinical application of TAMIS was first described in 2009 [35]. A hybrid between transanal endoscopic microsurgery (TEM), first described by Buess et al. in 1983 in an animal experiment, and single-port laparoscopy where a transanal multichannel port is used combined with conventional laparoscopic instruments [36].

Over the past years, considerable experience has been gained with the TAMIS technique for local excision of rectal neoplasia [37]. The improved visualization achieved with TAMIS has helped to expand the indications for this technique, for example in redo-surgery in IBD. Redo-surgery is often impeded by limited visualization due to adhesions, fibrosis, and distortion of the anatomical planes after anastomotic leakage. Early salvage of anastomotic leakage after IPAA can consist of surgical closure of the defect after Endosponge® therapy of the presacral cavity [38]. A highly effective novel technique without increasing costs.

**Fig. 1:**
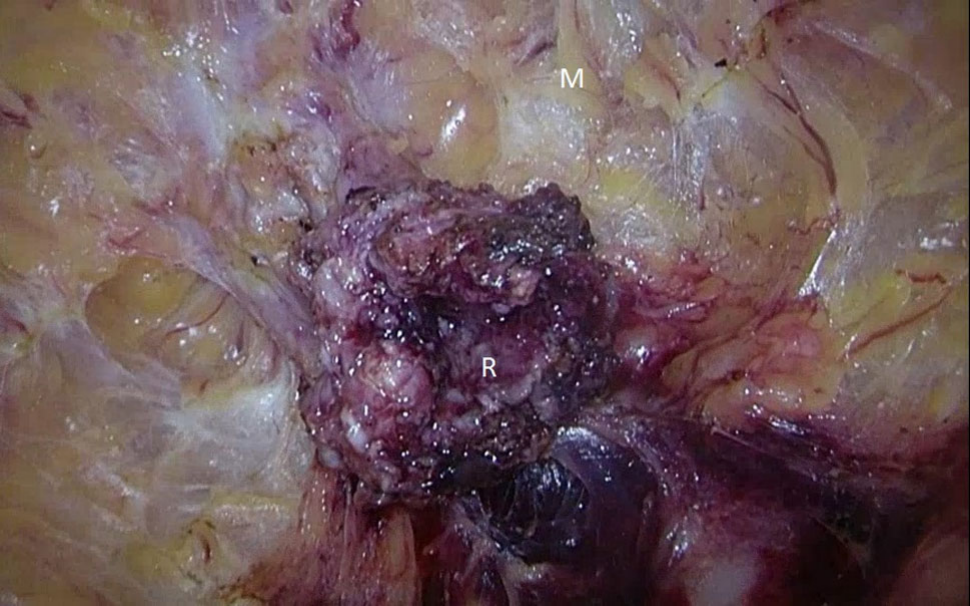
Proctocolectomy and pouch creation with TAMIS in a 50-year-old female patient with therapy refractory UC. Start of close rectal dissection (*M* mesorectal fat, *R* closed rectum)

**Fig. 2:**
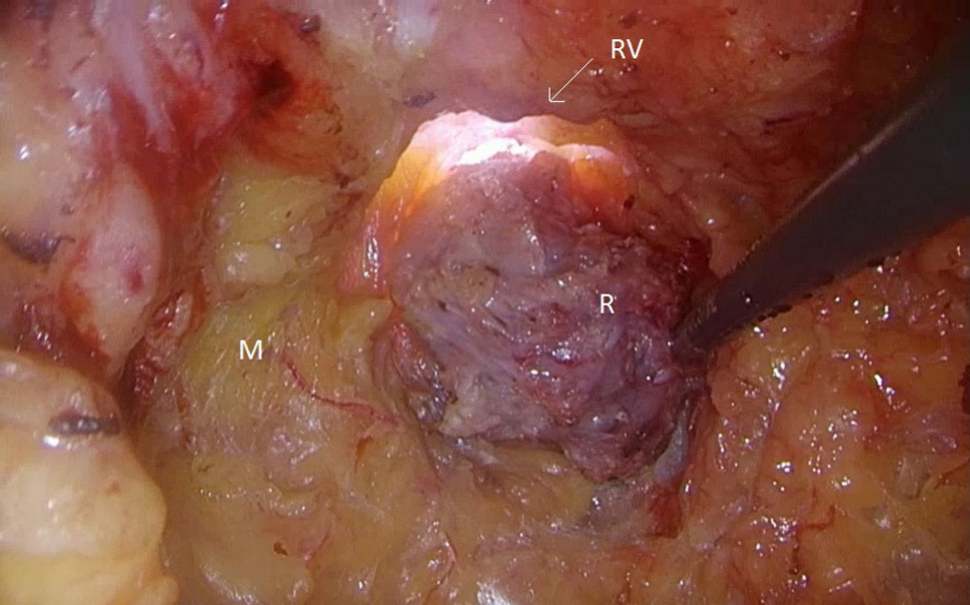
TAMIS bottom-up view after opening Douglas pouch with imminent rendez-vous (*M* mesorectal fat, *R* closed rectum, *RV* rendez-vous point)

**Fig. 3:**
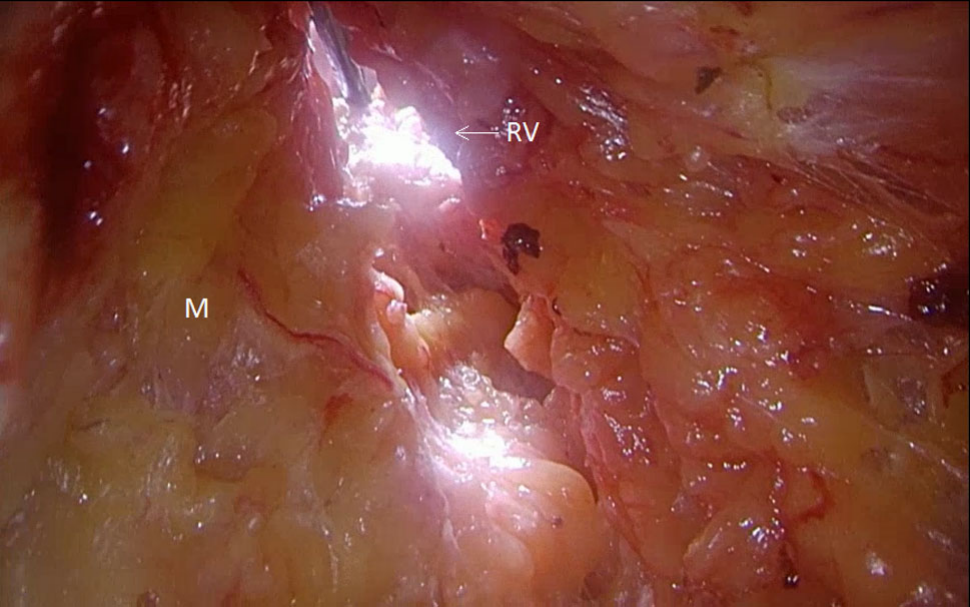
TAMIS bottom-up view after rendez-vous (*M* mesorectal fat, *RV* rendez-vous point)

**Fig. 4:**
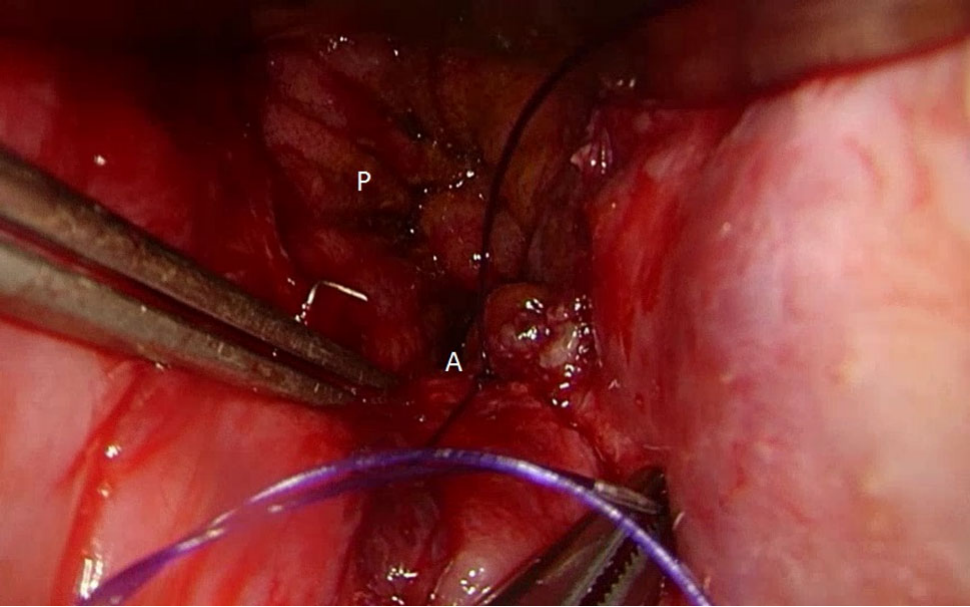
TAMIS over sewing the circular stapled ileoanal anastomosis (*P* pouch, *A* IPAA)

Particularly in patients with a narrow pelvis, the TAMIS approach with its ability to increase the mobilization of the rectum and improve visibility, may be valuable. The TAMIS technique is currently used in some cases in IBD (Figs. 1, 2, 3, 4). The first laparoscopic-assisted transanal colectomy for UC was reported in 2012 by Lacy et al. [39].

A novel single-port application is the TAMIS completion proctectomy, combining single or multi-port mobilization of mesentery, pouch creation, and top–down completion proctectomy, with TAMIS bottom-up completion proctectomy. Performing a close rectal dissection with a tailored transanally transection of the distal rectum is a very promising technique for a minimal invasive completion proctectomy. Lyanage et al. reported on nine IBD patients undergoing a transanal endoscopic completion proctectomy with a mean rectal stump of 17.8 cm (±6.1) [40]. In the patients where the peritoneal cavity was entered, there was no small-bowel injury. Another pilot study assessed the safety and feasibility of transanal rectal excision in fourteen patients of which nine with benign disease (one UC and one CD patient). The median duration of transanal surgery was 55 min (range 35–95) and there was minimal postoperative morbidity (median follow-up of 6.3 months, range 1.5–13.8) [40].

## Are there additional costs?

There is literature delineating that laparoscopic surgery induces additional costs when compared to open procedures. Nevertheless, these studies established that direct costs per case are significantly less for the laparoscopic surgery groups in CD [41, 42]. In single-port laparoscopic surgery the costs of disposable ports are added to the overall hospitalization costs. However, the average single post system costs do not way up to overall hospitalization costs of additional admission days [13]. Ahmed et al. reviewed the literature on cost-effectiveness of single-port laparoscopic surgery in abdominal and pelvic surgery and established that single-port procedures have secondary benefits including a decreased hospital stay, a faster return to work and improved cosmesis, with a possible positive effect on total costs [43]. Further research on this topic is needed.

## Advantages and disadvantages of multi-port laparoscopic surgery

There are several advantages for single-port laparoscopy when compared to multi-port laparoscopic surgery. With less incisions, a potential reduction of postoperative pain be accomplished with a reduction of morphine use [13]. In addition, there will naturally be fewer incisional hernias with single-port laparoscopy when compared to multi-port laparoscopic surgery. It is unlikely that a faster postoperative recovery and a potential shorter hospital stay can be expected particularly if patients are treated in an enhanced recovery program.

Superior cosmesis can be accomplished, due to port placement at the extraction site, future stoma site, or at the umbilicus. With close colon resection, the specimen size can be limited to allow transumbilical extraction. Some consider cosmesis of secondary importance, however patient satisfaction and body image are important patient-related outcomes possibly influencing quality of life [9, 44]. Transumbilical incision is advised for superior cosmesis. In case of a large inflammatory mass extraction via a Pfannenstiehl incision may be indicated.

It has to be emphasized that singe-port surgery is not suitable in all cases. The single port decreases the range of motion for the surgeon, since straight instruments have to be handled through a small single incision.

The downside of this might be a possible increase in parastomal hernia due to enlargement of the stoma site to facilitate the single port and the specimen extraction. Randall et al. observed a higher rate of parastomal hernias (18 %, *p* = 0.04) in patients after laparoscopic ostomy creation when compared to open surgery [45]. In addition, most parastomal hernias (60 %) during follow-up occurred in patients where the resection specimen was extracted through the stoma site. In 40 % of these patients, corrective surgery was required. Extraction of a bulky resection specimen via the ostomy site may weaken the supporting musculature.

## Conclusion

Operative techniques have evolved rapidly over the past decades. There is still limited literature on single-port laparoscopic surgery in IBD. However, several (matched) case series demonstrated that single-port laparoscopic surgery is a feasible and safe approach in IBD. There are several beneficial aspects with single-port laparoscopy with respect to postoperative pain, morphine use, length of hospital stay, and (functional) long-term outcomes, when compared to conventional multi-port laparoscopic surgery. Promising indications are ileocolic resections, (subtotal or procto) colectomy if the specimen is not too bulky. The TAMIS completion proctectomy in Crohn’s or completion proctectomy with IPAA are likewise promising developments that need to be studied further.

## Abbreviations

IBD: Inflammatory bowel disease
CD: Crohn’s disease
UC: Ulcerative colitis
IPAA: Ileal pouch-anal anastomosis
RCTs: Randomized controlled trials
IQR: Interquartile range
SD: Standard deviation
TAMIS: Transanal minimally invasive surgery
TEM: Transanal endoscopic microsurgery
